# Effect of Jaw Exercise on Mouth Opening and Trismus in Head and Neck Cancer Patients Undergoing Radiotherapy: A Randomised Controlled Trial

**DOI:** 10.3390/cancers18193215

**Published:** 2026-10-05

**Authors:** Tharmentheran Pitchaimuthu, Mohamad Yunus Mohd Razif, Khairiyiah Sidek, Syed Nabil

**Affiliations:** 1 Department of Oral and Maxillofacial Surgery, Faculty of Dentistry, Universiti Kebangsaan Malaysia, Jalan Raja Muda Abdul Aziz, Kuala Lumpur 50300, Malaysia; p138663@siswa.ukm.edu.my; 2 Department of Otorhinolaryngology, Faculty of Medicine, Universiti Kebangsaan Malaysia, Jalan Yaacob Latif, Kuala Lumpur 56000, Malaysia; razif72@ukm.edu.my; 3 Department of Radiotherapy, Hospital Canselor Tuanku Muhriz, Universiti Kebangsaan Malaysia, Jalan Yaacob Latif, Kuala Lumpur 56000, Malaysia; khayrea@gmail.com

**Keywords:** head and neck cancer, radiotherapy-induced trismus, maximal mouth opening/maximal interincisal distance, mandibular functionality

## Abstract

**Background/Objectives**: We aimed to assess the efficacy of a simple intervention by comparing changes in mouth opening and trismus status among HNC patients during radiotherapy treatment. It also explored its effect on the patient’s mandibular function impairment. **Methods**: This is a Randomised Controlled Trial in which subjects were divided into groups undergoing jaw exercise with a wooden tongue depressor versus a basic jaw exercise group. The primary outcome of this study was changes in maximal mouth opening (MMO)/maximal interincisal distance (MID) measured at baseline (before treatment), at the 10th and 20th week after initiation of radiotherapy. The secondary outcome included trismus prevalence and mandibular functionality, which were assessed in relation to trismus severity and the effectiveness of the intervention. **Results**: A total of 38 subjects were recruited, comprising 19 (50.0%) in the control group and 19 (50.0%) in the intervention group. There were 22 (57.9%) males and 16 (42.1%) females. Overall, the mean age of the patients was 52.5 years (22 to 74 years). Our clinical assessment showed no statistically significant differences (*p* = 0.835) in mouth opening among the groups, with an overall median of 0mm (IQR 7.0). **Conclusions**: Mouth opening did not worsen from baseline to 20 weeks after initiation of radiotherapy, and the addition of wooden tongue depressors did not confer a statistically significant benefit over basic jaw exercise alone. **Trial Registration**: ISRCTN96737564, registered on 26 August 2026, retrospectively registered.

## Simple Summary

Patients with head and neck cancer (HNC) are frequently exposed to various treatment-related complications, particularly those arising from radiotherapy or ablative surgery. Radiotherapy-induced trismus is one of the well-known complications. Despite advancements in radiotherapy technologies and techniques, trismus management remains inadequate. In this Randomised Controlled Trial, we investigated whether a structured wooden tongue depressor-assisted jaw exercise programme could improve mouth opening compared with basic jaw exercises in patients with head and neck cancer undergoing radiotherapy. We recruited and evaluated 38 patients using maximal mouth opening/maximal interincisal distance with a calliper and a questionnaire to assess mandibular impairment. Both groups showed small, non-significant changes in mouth opening across the follow-up period. Trismus was also significantly associated with greater mandibular functional impairment and patient-reported difficulty opening the mouth. These findings may support early jaw mobilisation during radiotherapy; however, the trial was limited by its small sample size, short-term follow-up, retrospective trial registration, and absence of a non-exercise control group. Larger, longer-term studies are needed to determine whether wooden tongue depressor provides additional benefit over basic jaw exercises alone.

## 1. Introduction

Head and neck cancer (HNC) represents a heterogeneous group of malignancies with a substantial disease burden, particularly in Southeast Asia, where tobacco use, alcohol consumption, betel quid chewing and genetic factors contribute to its development [1,2,3]. Radiation therapy (RT) serves as a crucial treatment modality for HNC. Despite improvements in RT delivery, treatment-related oral morbidity remains common. Fibrosis, xerostomia, dysphagia, mucositis and trismus can persist beyond treatment and may substantially affect nutrition, communication, oral hygiene and quality of life [4,5].

Trismus is generally defined in head and neck oncology as a maximum interincisal opening below 35 mm [6]. It may arise from tumour-related involvement of the masticatory apparatus, surgical scarring or radiotherapy-related injury to the muscles of mastication, temporomandibular joint and surrounding connective tissues [7,8]. Radiation-induced trismus (RIT) injury promotes inflammation, microvascular damage and progressive fibrosis, reducing tissue elasticity and jaw excursion. Dose to the masticatory structures has also been associated with subsequent trismus [9,10,11].

Exercise-based therapies have therefore been proposed to preserve mandibular range of motion. Available programmes include active jaw movements, passive stretching and the use of jaw-mobilising devices such as TheraBite Jaw Motion Rehabilitation System^®^ (Therabite), Dynasplint Trismus System^®^ (DTS) and wooden tongue depressors. However, studies differ considerably in exercise frequency, duration, starting time and choice of device, and evidence that one approach is superior remains limited [12,13]. Randomised studies of prophylactic exercise during RT have reported mixed results, with several showing no significant advantage over usual or active care [14,15,16].

Loorents et al. (2014), in a randomised study of 66 HNC patients, found no significant difference in maximum interincisal opening between patients undergoing intensive prophylactic TheraBite training and controls during RT or during follow-up [15]. Meanwhile, previous studies have suggested potential improvement in mouth opening using wooden tongue depressors [17,18]. 

The present study therefore evaluated a structured wooden tongue depressor programme initiated in patients undergoing RT for HNC. The primary objectives were to compare change in maximal mouth opening (MMO)/maximal interincisal distance (MID) between the intervention and active-control groups before and after RT. Secondary objectives were to assess mandibular function using a questionnaire and selected demographic and clinical factors associated with trismus. 

## 2. Methods

### 2.1. Study Design and Subject Recruitment

This Randomised Controlled Trial (RCT) investigates the effectiveness of a wooden tongue depressor as an exercise tool. It evaluates simple jaw exercises to alleviate trismus in HNC patients undergoing RT at Hospital Canselor Tuanku Mukhriz (HCTM). Patients were enrolled according to the pre-specified inclusion and exclusion and allocated to the control and intervention groups. The control group patients were instructed to do basic jaw exercises. In contrast, the intervention group were engaged in a comprehensive programme using wooden tongue depressors. Patients who met the following inclusion criteria were recruited: (a) age group above 18 years old; (b) coherent—demonstrates the capacity to perform mouth opening exercises independently and effectively; (c) able to communicate either in English, Bahasa Malaysia, or Mandarin; (d) patients diagnosed with head and neck cancer with documented tumour staging and histopathological examination excluding larynx; and (e) underwent radiotherapy treatment either exclusively or combined with chemotherapy and/or surgical treatment. The exclusion criteria were (a) poor general health with need of assistance or care and (b) patient is unable to perform mouth exercises independently.

### 2.2. Randomisation, Allocation Concealment, Blinding

Randomisation of the patients was done by a statistician from the department. Patients were randomised via block randomisation with a 1:1 allocation ratio using Random Allocation Software version 1.0, a computer-based programme designed to produce random allocation sequences for clinical and experimental studies [19]; no assessment of pre-existing trismus was performed prior to randomisation, and allocation was therefore not stratified by baseline trismus status. Upon randomisation by the statistician, the sequence was concealed from the investigators and participants in sequentially numbered, opaque, and sealed envelopes. The supervising investigator (S.N.), who was blinded to group allocation, performed the baseline MMO/MID measurement and all subsequent follow-up assessments. After completion of the baseline assessment, the principal investigator (T.P.), who enrolled the patients, opened the corresponding envelope. Patients allocated to the intervention arm were then taught the intervention training regimen by the principal investigator, while those allocated to the control group received the standard jaw-exercise therapy; the principal investigator also reinforced exercise adherence at follow-up visits. The principal investigator who performed the allocation and taught the exercise regimen was not involved in outcome assessment. This study was single-blinded: assessor blinded, with the assessor performing the MMO/MID measurements was unaware of the specific intervention each participant received.

### 2.3. Sample Size Determination

The sample size calculation was performed using G*Power software version 3.1, with an α-significance level of 0.05 and a statistical power of 95%. The calculation was based on data from a previously published randomised clinical trial [18]. Based on the reported means and standard deviations of maximal interincisal opening between the intervention and control groups in that study [18] (effect size d = 1.25, α = 0.05, power = 95%), the minimum required sample size was 30 participants (15 per group). To accommodate a potential attrition rate of 20%, a total of 36 participants were planned for recruitment. Participants were randomly assigned in a 1:1 ratio to either the intervention or active control group.

### 2.4. Statistical Analysis

Statistical analysis was performed using SPSS software (Version 29.0). Normality testing showed that the samples were not normally distributed (*p* < 0.05). The distributions of the change scores within each treatment group were also visually inspected using histograms and normal Q-Q plots to assess skewness, outliers and departures from normality. Therefore, we performed non-parametric tests for all data, using the Shapiro–Wilk test as an indicator, as the sample size was <50. Categorical variables were compared between groups using Fisher’s Exact test. For continuous outcomes, the Mann–Whitney U test was used to compare the change in MMO/MID (and MFIQ score) between the intervention and control groups at each time interval: ΔT2 = T2 − T1 and ΔT3 = T3 − T1. The null hypothesis tested was that the distribution of change scores did not differ between the intervention and control groups. The Wilcoxon signed-rank test was used to compare, within each treatment group, the paired change scores at the two follow-up assessments (ΔT2 = T2 − T1 versus ΔT3 = T3 − T1). The null hypothesis tested was that the median of the paired differences (ΔT3 − ΔT2, equivalent to T3 − T2) was zero, indicating no within-group differences in MMO/MID between the two follow-up assessments. The test result was considered statistically significant if the *p*-value < 0.05. Analyses followed the complete-case principle. All available outcome data were included in the analyses. Missing data were not imputed; analyses at each follow–up time point were therefore based on the available observations within the original randomised groups. All secondary analysis *p*-values are reported as unadjusted nominal *p*-values. These analyses are exploratory and interpreted as hypothesis-generating rather than confirmatory.

### 2.5. Data Collection

Assessments were conducted at three time points: before RT (T1), 10 weeks after initiation of RT (T2) and 20 weeks after initiation of RT (T3). Demographic variables included age, sex, ethnicity, type of cancer, treatment regimen, Tumour–Node–Metastasis (TNM) staging, RT dose (Gy), RT cycle were recorded at T1. Clinical variables MMO/MID and mandibular function impairment questionnaire (MFIQ) score were collected longitudinally (T1–T3).

### 2.6. Mouth Opening Exercise

The active control group received basic jaw-exercise instructions consisting of mouth opening and closing with side-to-side jaw movement. Participants were instructed to perform the exercise twice daily for 30 s throughout the 20-week study period. Participants were not instructed to maintain a static position during any individual movement.

The intervention group underwent a structured 20-week wooden tongue depressor based on a 5-5-30 protocol: five exercise sessions per day, five cycles per session and a 30-s passive stretch during each cycle. Each session began with ten repetitions of jaw opening and small lateral movement without the tongue depressors. Participants then moved the chin to the left, right and forward, holding each position for 3 s, followed by maximal mouth opening held for 10 s. Following this, passive stretching was performed with a stack of wooden tongue depressors inserted to the maximum tolerable opening. One additional depressor was then added and the stretch was maintained for 30 s. The number of wooden tongue depressors was increased gradually based on each participant’s tolerance, without a predefined step-up algorithm. This approach facilitated progressive passive stretching. The regimen was reinforced during scheduled follow-up visits; adherence was not quantitatively measured.

### 2.7. Outcome Measures

The primary outcome measured was the change from baseline in MMO/MID. All MMO/MID measurements were performed by the supervising investigator (S.N.), an oral and maxillofacial surgeon, using a calibrated digital WIHA calliper with the participant seated upright. MID was defined as the maximal vertical distance between the incisal edges of the maxillary and mandibular incisors. Where incisal measurement was not applicable, MMO was measured between the maxillary and mandibular alveolar ridges. Values were recorded in millimetres. Participants with MMO/MID ≤35 mm were classified as having trismus [6].

The trismus prevalence and mandibular functionality using MFIQ were used to assess trismus-related symptoms for the secondary outcome. Patients completed this questionnaire through an interview by the doctor. This MFIQ comprises 17 items encompassing the difficulty of chewing hard and soft foods, taking large bites, laughing, eating certain foods, engaging in social activity and speaking. Items were scored on a 5-point Likert scale (1–5), recoded to a 0–4 scale for analysis, where 0 denotes no difficulty and 4 denotes the greatest difficulty. The total MFIQ score for each participant was calculated as the sum of the 17 recoded item scores, giving a possible range of 0 to 68, with a higher score denoting more serious functional impairment. Dividing the total score by 68 yields an overall score ranging from 0 to 1, for which a value of ≤0.3 denotes a low degree, >0.3–0.6 a moderate degree, and >0.6 a high degree of functional impairment. This scale has demonstrated good test–retest reliability (r = 0.80) and internal consistency (Cronbach’s α between 0.63 and 0.95) when used with temporomandibular joint disorders [20].

### 2.8. Ethical Consideration

Ethical approval for this study was obtained from the Research Ethics Committee of Universiti Kebangsaan Malaysia (protocol code: UKM PPI/111/8/JEP-2023-524). The study was conducted in accordance with the Declaration of Helsinki. The trial was retrospectively registered with the ISRCTN registry (ISRCTN96737564) on 26 August 2026 because the requirement for prospective registration was inadvertently overlooked at study initiation; the omission was identified only during manuscript preparation, and the trial was registered immediately thereafter. This delay was administrative and did not involve any change to the approved protocol. The primary outcome, key secondary outcome, data collection procedures, sample-size calculation, and eligibility criteria reported in this manuscript were pre-specified in the ethics-approved study protocol before participant recruitment began and are consistent with the trial registry, with one exception: quality of life, listed in the registry as an exploratory objective, was not measured in this study.

## 3. Results

Overall, 43 patients were initially enrolled in this study. Following initial screening using the predetermined inclusion and exclusion criteria, five patients were excluded. Of these, four patients presented with laryngeal carcinoma, and one patient declined radiotherapy treatment. Consequently, all 38 randomised patients were retained in their originally assigned groups. During the follow-up period, three patients in the intervention group dropped out: one patient had to suspend radiotherapy, one patient passed away, and one patient declined to continue participation. In the control group, one patient declined to continue participation during follow-up. As a result, follow-up analyses were based on 34 patients with available data, while all patients remained classified according to their randomised allocation (Figure 1).

The mean age of the 38 patients was 52.5 ± 14.8 years, ranging from 22 to 74 years. A total of 22 participants (57.9%) were male and 16 (42.1%) were female. The majority were Malay (65.8%), while 31.6% were Chinese, with one Indigenous participant. A total of 18 patients (47.4%) were diagnosed with nasopharyngeal carcinoma (NPC), whereas 20 (52.6%) had non-NPC head and neck malignancies. In this study, non-NPC patients were further subdivided into the following categories: oral and oropharyngeal carcinoma/angiosarcoma, salivary gland carcinoma, paranasal and temporal bone carcinoma. Supplementary Table S1 summarised the distribution of non-NPC primary tumour sites by study group. The overall mean radiotherapy dose was 64.2 ± 8.7 Gy. The mean age was 49.2 ± 16.6 years in the intervention group and 55.8 ± 12.4 years in the control group (*p* = 0.243). The majority of patients in the intervention group received intensity-modulated radiation therapy (IMRT) ± chemotherapy. Among these patients, 42.1% were classified as stage 4 and 31.6% as stage 3 according to TNM staging. In contrast, the control group underwent surgery + IMRT ± chemotherapy the most. The majority of tumours in this group were classified as stage 4 according to TNM staging. No statistically significant between-group differences were detected in measured baseline characteristics (Table 1 and Table 2).

### 3.1. Effects on MMO/MID and Effectiveness of Trismus Device

From T2 to T1, the median change in MMO/MID was 0.5 mm (IQR 6.5) in the intervention group and −1.0 mm (IQR 4.3) in the control group (mean ± SD: 0.1 ± 5.8 mm and −0.5 ± 4.5 mm, respectively). From T3 to T1, the median changes were 0.0 mm (IQR 7.8) and 0.5 mm (IQR 6.3) in the intervention and control groups, respectively (mean ± SD: 0.1 ± 6.2 mm and −0.2 ± 6.1 mm). The difference was not statistically significant, either between groups (*p* = 0.406 at T2 − T1 and *p* = 0.835 at T3 − T1) or within each group between the two follow-up time points (*p* = 0.748 for the intervention group and *p* = 0.343 for the control group) (Table 3).

Using MMO/MID ≤35 mm as the cut-off point for trismus, at T1, six (31.6%) patients in the intervention and three (15.8%) patients in the control had trismus. At T2, the total number of patients who presented with trismus was four (11.8%). In the control group, the total number of patients with trismus was three (16.7%), while the intervention group had one (6.3%) patient with trismus. The absolute risk difference (ARD) was −10.4%, indicating an observed prevalence that was 10.4% lower in the intervention group. At T3, the intervention group had two (12.5%) patients with trismus, while the control group remained unchanged at three (16.7%), corresponding to an ARD of -4.2%. No statistically significant between-group difference was observed in mouth opening or trismus prevalence by T3 (Table 4). However, given the modest sample size, randomisation may not have fully balanced all potentially relevant prognostic factors between the groups. Among patients with baseline trismus, two (33.3) patients in the intervention group no longer met the trismus criterion at follow-up, whereas all three (100.0) control group patients remained classified as having trismus. Meanwhile, among those without baseline trismus, one (7.7%) in the intervention group developed trismus by T3, while no new cases were reported in the control group.

### 3.2. Effects on MFIQ

When analysis was done between groups, the MFIQ scores did not differ significantly at any time point. However, when patients were grouped according to the presence of trismus, MFIQ scores were consistently higher among those with trismus at every time frame. The difference was statistically significant at T1 and T2 (17.0 ± 16.3 vs. 2.67 ± 5.5; *p* = 0.001), (15.3 ± 6.5 vs. 3.3 ± 5.4; *p* = 0.002) and at T3, this difference was not statistically significant (10.4 ± 8.2 vs. 4.6 ± 8.0; *p* = 0.097) (Table 5).

### 3.3. Association Between Demographic and Clinical Variables Affecting Trismus

No significant association was observed between age and trismus during follow-up. Cancer type was associated with trismus at baseline, where eight of the nine participants with trismus had non-nasopharyngeal cancer (*p* = 0.021), but the association was not statistically significant at T2 or T3. At T2, surgical status demonstrated a statistically significant association with trismus (*p* = 0.039). However, this association was not observed at T3. Details of the surgical procedures performed among patients who underwent surgery are summarised in Supplementary Tables S2 and S3. Furthermore, the TNM stage did not show a significant correlation with trismus in the analyses conducted within the study. Radiotherapy dose was not significantly associated with trismus at T2 or T3. Nevertheless, the study noted that participants who developed or retained trismus after RT tended to have received higher total doses than those without trismus. Table 6 summarises the relationship between demographic and clinical variables affecting trismus in each timeline.

## 4. Discussion

This RCT evaluated whether a structured wooden tongue-depressor-assisted jaw exercise programme provided additional benefit over basic jaw exercises in maintaining mouth opening among patients with head and neck cancer undergoing RT. The main finding was that changes in MMO/MID did not differ significantly between the intervention and control groups at either 10 or 20 weeks after RT. Meanwhile, the absence of a statistically significant between-group difference in MMO/MID is consistent with previous randomised trials evaluating prophylactic exercise for RIT. Loorents et al. (2014), Høgdal et al. (2015), and Bragante et al. (2020) also observed no significant differences in mouth opening between patients engaging in structured prophylactic exercise and those receiving either usual care or less intensive exercise [14,15,16]. These protocol findings underscore the inherent challenges in demonstrating the superiority of one exercise regimen over another, particularly when the control group was immobilised with active jaw mobilisation. In the present study, the control group was not untreated; instead, participants performed regular opening, closing, and lateral jaw exercises for a duration of 20 weeks. This should be kept in mind when interpreting the null result: it should not be read as evidence that exercise itself confers no benefit, but rather that the additional effect of the more intensive wooden-tongue-depressor-based exercise protocol relative to the basic jaw-exercise regimen appears modest and was not detected in this small sample. Analysis of difference from baseline (T1) provided additional insight into the clinical pattern observed. The present analysis focused on the difference in mouth opening relative to each patient’s baseline, rather than relying exclusively on absolute mean MMO/MID values at specific time points. This approach may be advantageous because such discrepancies can obscure data interpretation and ultimately affect the assessment of the actual difference in outcomes. Unfortunately, no previous studies have analysed the data this way to allow direct comparison.

The observed pattern of greater improvement at 10 weeks, followed by a smaller difference at 20 weeks, likely reflects the dynamic biological processes that occur after RT. Consistent with findings by Barañano et al. (2011), who reported that gains from jaw mobilisation are most notable in the early weeks of therapy and tend to plateau over time [21], our study also demonstrated the greatest improvement in the intervention group at the 10-week assessment, followed by a minimal change at 20 weeks. One possible explanation is that the combined effect of the more intensive exercise protocol with mechanical stretching may exert its greatest effect during the initial phase of tissue injury, before progressive radiation-induced fibrosis (RIF) becomes established. As fibrosis matures, collagen deposition, tissue contraction, and reduced elasticity may progressively limit the response to stretching.

Wooden tongue depressors were selected because of their simplicity, availability, and relatively low cost. Various mechanical devices, including Therabite and DTS, have been investigated for trismus rehabilitation. Melchers et al. (2009) reported potential benefit with TheraBite; however, their study was not randomised or controlled [22]. Lee et al. (2018) subsequently compared TheraBite with a wooden tongue depressor and found improvement in mouth opening with both interventions but no significant superiority of either device [17]. Their study also demonstrated a substantial difference in device cost, with wooden spatulas being considerably less expensive. This is particularly relevant in resource-constrained healthcare environments, where the availability and affordability of rehabilitation devices may influence implementation. Nevertheless, availability and affordability alone do not ensure successful clinical implementation. The programme requires repeated exercises and progressive passive stretching over an extended period, which may impose a burden on patients. Furthermore, treatment-related pain, mucositis, fatigue, and other adverse effects may further reduce tolerance and adherence during RT. Meanwhile, a study by Wang et al. (2019) reported a considerably greater improvement in MID following an intervention incorporating tongue depressors [18]. However, direct comparison with the present study is difficult because their intervention consisted of multiple components, including active exercise, passive stretching, warm compression, masseter massage, and weekly telephone support. Consequently, the observed improvement could not be attributed solely to the tongue depressor. Their study also emphasised the potential importance of adherence and ongoing professional reinforcement. In the present study, patients were given exercise instructions and the regimen was reinforced during scheduled reviews, but adherence was not objectively monitored. This may partly explain the relatively modest difference observed between groups. Meanwhile, transition analysis in trismus status suggested a possible reduction in trismus among patients in the intervention group, whereas the control group patients with baseline trismus remained classified as having trismus at both follow-up assessments. Although these findings may indicate a potential benefit of the wooden tongue depressor programmes with patients with pre-existing trismus, this should be interpreted cautiously. Several factors may limit the strength of this interpretation. Changes in mouth opening may also have been influenced by tumour response, postoperative healing, rehabilitation or exercises performed by patients in both groups. Accordingly, these results provide a sign that the intervention may help reduce persistent trismus, but they do not establish a causal treatment effect. Moreover, given the very small number of patients with baseline trismus in each group (six in the intervention group and three in the control group), this transition analysis is markedly underpowered; these observations should therefore be regarded as hypothesis-generating only and should not be interpreted as evidence of intervention efficacy within this subgroup. Predefined adherence monitoring and appropriate adjustment for clinical and treatment-related factors are needed to determine whether the wooden tongue-depressor program improves trismus outcomes in future studies.

Moreover, an important finding of this study was the consistent relationship between objectively defined trismus and MFIQ. Patients with trismus had significantly higher MFIQ scores than those without trismus at baseline and at 10 weeks. This association was no longer statistically significant at 20 weeks (*p* = 0.097), although patients with trismus continued to show numerically higher MFIQ scores at this time point. These findings indicate that a reduction in MMO/MID is not merely an anatomical measurement but corresponds to meaningful limitations in mandibular function, particularly in the earlier post-radiotherapy period. The MFIQ evaluates activities including chewing, biting, speaking, laughing, and other mandibular functions, suggesting that patients with restricted mouth opening experience a broader functional burden in daily life. Interestingly, total MFIQ scores did not differ significantly between the intervention and control groups despite the numerical improvement in MMO/MID observed in the intervention arm. Similar observations have been reported in previous studies, including Kamstra et al. (2016), in which improvements in mouth opening did not necessarily translate into comparable improvements in overall mandibular function [23]. This discrepancy may reflect the multidimensional nature of mandibular function. Although mechanical stretching may increase the anatomical range of mouth opening, other functional problems related to chewing, speech, pain, muscular weakness, tissue fibrosis, and neuromuscular adaptation may persist.

Several demographic and clinical factors were evaluated for their potential association with trismus. Although increasing age has been associated with reduced joint mobility and temporomandibular function [24], no significant association between age and trismus was observed in our study. In contrast, Van der Geer et al. (2019) reported a significant association (*p* = 0.01), which may partly reflect their substantially larger sample size (*n* = 730) and greater statistical power [25]. Cancer type was significantly associated with trismus at baseline (T1), with eight of nine patients with trismus having non-nasopharyngeal carcinoma. This supports previous findings that tumours involving or located close to the masticatory space and infratemporal fossa are more likely to impair mouth opening [7,8]. However, this association was not observed at later assessments, possibly due to the smaller number of evaluable patients during follow-up. No significant association was found between tumour size and trismus. In contrast, Wetzels et al. (2014) identified tumour size as an independent predictor in patients with oropharyngeal cancer, suggesting that anatomical tumour location may be more important than tumour size alone [26]. Surgery, particularly extensive resection and flap reconstruction, has also been associated with trismus [25,27]. In the present study, surgical status was significantly associated with trismus at T2; however, this association was no longer statistically significant at T3. This transient association may reflect the early effects of postoperative scarring, tissue contracture, and flap-related restriction, followed by gradual healing and functional adaptation. Nevertheless, the absence of significance at T3 should be interpreted cautiously, particularly given the small sample size. RT remains an important contributor to trismus through progressive inflammation and fibrosis of the masticatory tissues [28]. Although total RT dose was not significantly associated with trismus at T2 or T3, patients who developed trismus tended to receive higher radiation doses. These findings should be interpreted cautiously. The total prescribed dose does not reflect the actual dose received by individual components of the masticatory apparatus. Previous studies have demonstrated dose-dependent effects on specific masticatory muscles. Kraaijenga et al. (2019) reported that doses exceeding 58 Gy to the lateral pterygoid and 22 Gy to the masseter were predictive of trismus, while Hague et al. (2018) found that doses above 40 Gy to ipsilateral masticatory structures were associated with trismus at six months [9,29]. These findings suggest that dose distribution to individual masticatory muscles may be more clinically relevant than total prescribed radiation dose.

Several limitations should be acknowledged. First, this trial was also registered retrospectively, which is a recognised threat to transparency because it precludes independent verification that the reported outcomes were not selected or modified after the results were known. As with any retrospectively registered study, the results should be interpreted with the awareness that selective outcome reporting cannot be entirely excluded. However, any deviation from the original protocol for this study has been disclosed in Section 2 to mitigate this concern. Second, this was a single-centre study with a relatively small sample, limiting statistical power and generalizability. The a priori sample-size calculation was based on a large effect size (d = 1.25) derived from a previous postoperative oral-cancer rehabilitation trial whose population and multicomponent intervention differed from the present trial, which may have overestimated the effect expected from adding wooden tongue depressors to basic jaw exercises in patients undergoing radiotherapy. With 34 participants included in the available-case analysis (16 intervention, 18 control), the study had 95% power to detect only a standardised between-group effect of approximately d = 1.28. Consequently, the study may have been unable to detect small to moderate between-group effects, increasing the risk of a type II error. Larger trials are required to provide more precise estimates and determine whether the observed differences represent a genuine intervention effect. Third, four participants were unavailable for assessments at T2 and T3. Given the small overall sample, even limited attrition may influence estimates of treatment effect. Fourth, despite randomisation, important baseline clinical heterogeneity remained between the study groups with respect to gender, tumour site, stage, surgical treatment, chemotherapy, and RT exposure. Therefore, each of these factors may independently affect MMO/MID and trismus risk. The observed outcomes may therefore reflect a combination of the exercise regimen and underlying clinical differences between patients. Fifth, the 20-week follow-up captures relatively early functional outcomes. RIF may continue to evolve beyond this interval, and trismus may become more apparent 6–12 months or longer after treatment. These findings are therefore limited to early post-RT outcomes and should not be interpreted as evidence of long-term benefit against RIF; longer follow-up is required to establish durable treatment effects. Sixth, adherence to the home exercise programme was not objectively monitored using an exercise diary or electronic monitoring method. Consequently, the magnitude of adherence in either group remains unknown. Any between-group imbalance in exercise frequency or protocol fidelity could not be assessed and may have biased the observed treatment effect estimates. Seventh, participant and intervention-provider blinding was not feasible. This may have introduced performance bias by influencing participants’ expectations, motivation, exercise intensity, adherence, or use of additional exercises. Although the blinded outcome assessor reduced the risk of detection bias in MMO/MID measurements, assessor blinding could not eliminate these potential sources of performance bias. Eighth, population-specific validation of the MFIQ in HNC patients treated with RT is lacking. Although the MFIQ has been used in HNC-related trismus research, its reliability and validity have not been formally established in this population. The MFIQ results in this study should therefore be interpreted as exploratory, and future studies should assess the questionnaire’s psychometric properties in RT-treated HNC populations. Ninth, multiple secondary and subgroup analyses were performed across several time points without adjustment for multiplicity. Consequently, all secondary-analysis *p*-values should be regarded as exploratory, unadjusted estimates rather than confirmatory findings, and the associated secondary conclusions should be interpreted as hypothesis-generating only. Finally, total radiation dose does not adequately characterise exposure of the masticatory apparatus. Structure-specific dosimetric data, including dose-volume parameters for the masseter muscles, medial and lateral pterygoid muscles, temporomandibular joints, mandible, and other relevant components of the masticatory apparatus, were unavailable for this study. Future trials should integrate RT treatment-planning data with functional outcomes, and voxel-level dose mapping should also be considered to identify anatomical regions in which radiation exposure is most strongly associated with changes in MMO/MID. Despite these limitations, the study provides clinically useful evidence in which RIT research remains limited. It also demonstrates the feasibility of incorporating an inexpensive wooden tongue depressor protocol within routine HNC supportive care.

## 5. Conclusions

RIT is a severe complication in patients with HNC undergoing RT. Preserving mouth opening and reducing the impact of RIT are important for improving patient outcomes and quality of life. In this trial, mean mouth opening did not worsen from baseline to 10 and 20 weeks after initiation of RT in either the wooden tongue depressor or basic jaw exercise group. When comparing the two exercise approaches directly, no statistically significant between-group superiority was observed with the wooden tongue depressor exercises at either time point. Overall, early implementation of jaw exercises, with or without adjunctive devices, was associated with preservation of mouth opening among patients with HNC, although a causal effect cannot be established in the absence of a non-exercise comparator. These findings may inform clinical discussions about incorporating jaw exercise programmes for patients undergoing RT for HNC, regardless of whether adjunctive devices are included, as part of routine supportive care for RIT.

## Figures and Tables

**Figure 1:**
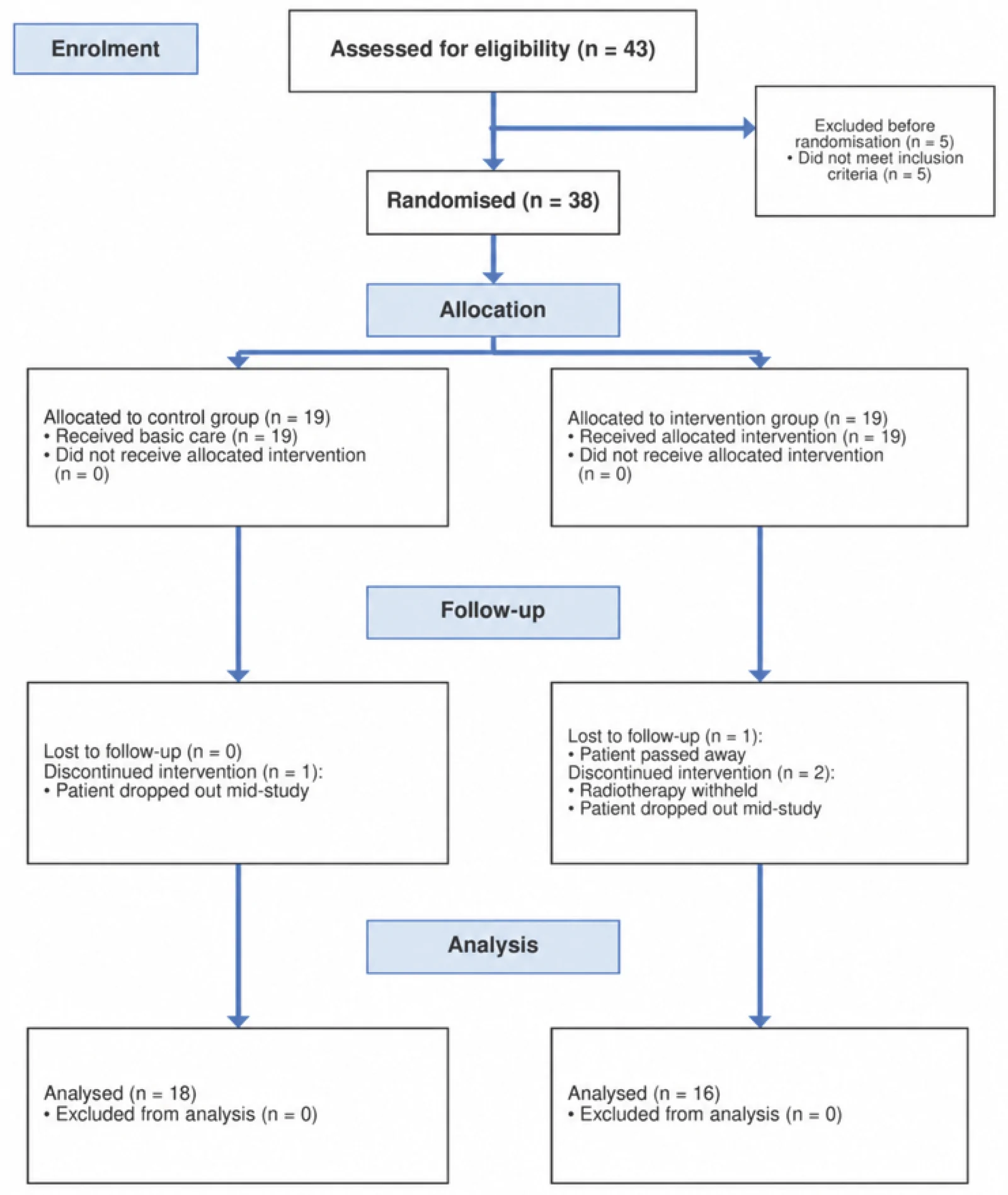
Flow chart of the study conduct.

**Table 1:** The demographic information of intervention and control groups.

No. of Patients, *n* (%)				
Demographic Information	Intervention 19 (100.0)	Control 19 (100.0)	Total 38 (100.0)	*p*-Value
Age (years)				0.243 ^a^
Mean ± Std	49.2 ± 16.6	55.8 ± 12.4	52.5 ± 14.8	
Median (IQR)	49.0 (34.0)	57.0 (17.0)	54.0 (25.0)	
Min	22.0	25.0	22.0	
Max	74.0	72.0	74.0	
Gender:				0.099 ^b^
Male	8 (42.1)	14 (73.7)	22 (57.9)	
Female	11 (57.9)	5 (26.3)	16 (42.1)	
Race:				0.170 ^b^
Malay	10 (52.6)	15 (78.9)	25 (65.8)	
Chinese	8 (42.1)	4 (21.1)	12 (31.6)	
Indigenous	1 (5.3)	0 (0)	1 (2.6)	

^a^ Mann–Whitney test; ^b^ Fisher’s Exact test.

**Table 2:** The clinical information of intervention and control groups.

No. of Patients, *n* (%)				
**Clinical Information**	**Intervention** **19 (100.0)**	**Control** **19 (100.0)**	**Total** **38 (100.0)**	***p*-Value**
Trismus Severity:				
Baseline (T1)				0.447 ^b^
Trismus	6 (31.6)	3 (15.8)	9 (23.7)	
No Trismus	13 (68.4)	16 (84.2)	29 (76.3)	
10th Week after RT (T2)				0.447 ^b^
Trismus	1 (5.3)	3 (15.8)	4 (10.5)	
No Trismus	15 (78.9)	15 (78.9)	30 (78.9)	
Default	3 (15.8)	1 (5.3)	4 (10.5)	
20th Week after RT (T3)				0.737 ^b^
Trismus	2 (10.5)	3 (15.8)	5 (13.2)	
No Trismus	14 (73.7)	15 (78.9)	29 (76.3)	
Default	3 (15.8)	1 (5.3)	4 (10.5)	
Type of Cancer:				0.142 ^b^
Nasopharyngeal Carcinoma	9 (47.4)	9 (47.4)	18 (47.4)	
Non-Nasopharyngeal Carcinoma	10 (52.6)	10 (52.6)	20 (52.6)	
Treatment Regimen:				0.226 ^b^
Surgery + IMRT ± Chemotherapy	5 (26.3)	9 (47.4)	14 (36.8)	
Surgery + 3DCRT + Chemotherapy	3 (15.8)	1 (5.3)	4 (10.5)	
IMRT ± Chemotherapy	11 (57.9)	7 (36.8)	18 (47.4)	
3DCRT ± Chemotherapy	0 (0)	2 (10.5)	2 (5.3)	
TNM Staging:				0.371 ^b^
Stage 1	2 (10.5)	3 (15.8)	5 (13.2)	
Stage 2	3 (15.8)	2 (10.5)	5 (13.2)	
Stage 3	6 (31.6)	3 (15.8)	9 (23.6)	
Stage 4	8 (42.1)	11 (57.9)	19 (50.0)	
RT (Gy)				0.606 ^a^
Mean ± Std	65.6 ± 5.5	62.8 ± 10.9	64.2 ± 8.7	
Median (IQR)	70.0 (10.0)	66.0 (10)	66.0 (10.0)	
Min	52.0	30.0	30.0	
Max	70.0	70.0	70.0	
RT (cycle)				0.438 ^a^
Mean ± Std	32.5 ± 3.7	31.1 ± 6.9	31.8 ± 5.5	
Median (IQR)	35.0 (5.0)	33.0 (5.0)	34.0 (5.0)	
Min	20.0	10.0	10.0	
MFIQ T1 (total score)				0.843 ^a^
Mean ± Std	8.1 ± 13.9	4.1 ± 6.3	6.1 ± 10.9	
Median (IQR)	0 (15.0)	1.0 (7.0)	0 (8.5)	
Min	0	0	0	
Max	50.0	21.0	50.0	
	**Intervention** **16 (100.0)**	**Control** **18 (100.0)**	**Total** **34 (100.0)**	
MFIQ T2 (total score)				0.416 ^a^
Mean ± Std	4.5 ± 7.7	4.9 ± 5.8	4.7 ± 6.7	
Median (IQR)	0 (5.5)	2.0 (9.8)	1.5 (9.0)	
Min	0	0	0	
Max	24.0	16.0	24.0	
MFIQ T3 (total score)				0.816 ^a^
Mean ± Std	5.6 ± 9.4	5.3 ± 7.2	5.4 ± 8.2	
Median (IQR)	0 (10.0)	0 (13.3)	0 (12.3)	
Min	0	0	0	
Max	28.0	22.0	28.0	

^a^ Mann–Whitney test; ^b^ Fisher’s Exact test.

**Table 3:** The comparison of the difference in mouth opening (mm) (T2–T1 vs T3–T1) between intervention and control groups.

Radiotherapy Timeline	Difference Mouth Opening (mm)	No. of Patients, *n* (%)			* *p*-Value	
		Intervention 16 (100.0)	Control 18 (100.0)	Total 34 (100.0)		
T2–T1	Mean ± Std	0.1 ± 5.8	−0.5 ± 4.5	−0.2 ± 5.1	120.00 (0.406)	
	Median (IQR)	0.5 (6.5)	−1.0 (4.3)	−0.5(6.0)		
	Min	−10.0	−5.0	−10.0		
	Max	14.0	11.0	14.0		
T3–T1	Mean ± Std	0.1 ± 6.2	−0.2 ± 6.1	−0.03 ± 6.1	138.00 (0.835)	
	Median (IQR)	0 (7.8)	0.5 (6.3)	0 (7.0)		
	Min	−14.0	−13.0	−14.0		
	Max	15.0	11.0	15.0		
Within-group *p*-value † (T2 − T1 vs. T3 − T1)					−0.345 (0.748)	−0.987 (0.343)

* Mann–Whitney U test (between-group comparison at each time point); † Wilcoxon signed-rank test (within-group comparison of paired signed change scores between T2 and T1 vs. T3 and T1).

**Table 4:** Trismus prevalence in the intervention and control groups at T1, T2, and T3.

**Radiotherapy Timeline**	**Trismus Severity**	**No. of Patients, *n* (%)**						* *p*-Value
		**Intervention** **19 (100.0)**		**Control** **19 (100.0)**		**Total** **38 (100.0)**		
T1	Trismus	6 (31.6)		3 (15.8)		9 (23.7)		0.447
	No trismus	13 (68.4)		16 (84.2)		29 (76.3)		
		**Intervention** **16 (100.0)**		**Control** **18 (100.0)**		**Total** **34 (100.0)**		
T2	Trismus	1 (6.3)		3 (16.7)		4 (11.8)		0.604
	No trismus	15 (93.8)		15 (83.3)		30 (88.2)		
T3	Trismus	2 (12.5)		3 (16.7)		5 (14.7)		1.000
	No trismus	14 (87.5)		15 (83.3)		29 (85.3)		
	**Baseline Status**	**No of Patients, *n* (%)**						
		**T1**	**T2: Trismus**	**T2:** **No Trismus**	**T2: Missing**	**T3: Trismus**	**T3:** **No Trismus**	**T3: Missing**
Intervention	Trismus	6 (100.0)	1 (16.7)	2 (33.3)	3 (50.0)	1 (16.7)	2 (33.3)	3 (50.0)
	No Trismus	13 (100.0)	0 (0)	13 (100.0)	0 (0)	1 (7.7)	12 (92.3)	0 (0)
Control	Trismus	3 (100.0)	3 (100.0)	0 (0)	0 (0)	3 (100.0)	0 (0)	0 (0)
	No Trismus	16 (100.0)	0 (0)	15 (93.8)	1 (6.3)	0 (0)	15 (93.8)	1 (6.3)

* Fisher’s Exact test.

**Table 5:** The relationship between trismus severity and mandibular function using MFIQ.

**Radiotherapy Timeline**	**MFIQ Score**	**No. of Patients, *n* (%)**			* *p*-Value
		**Trismus** **9 (23.7)**	**No Trismus** **29 (76.3)**	**Total** **38 (100.0)**	
T1	Mean ± Std	17.0 ± 16.3	2.67 ± 5.5	6.1 ± 10.9	50.00 (0.001) *
	Median (IQR)	14.0 (22.5)	0 (3.0)	0 (8.5)	
	Min	0	0	0	
	Max	50.0	21.0	50.0	
**Radiotherapy Timeline**	**MFIQ Score**	**No. of Patients, *n* (%)**			* *p*-Value
		**Trismus** **4 (11.8)**	**No Trismus** **30 (88.2)**	**Total** **34 (100.0)**	
T2	Mean ± Std	15.3 ± 6.5	3.3 ± 5.4	4.7 ± 6.7	9.50 (0.002) *
	Median (IQR)	14.0 (12.3)	0.5 (4.3)	1.5 (9.0)	
	Min	9.0	0	0	
	Max	24.0	19.0	24.0	
**Radiotherapy Timeline**	**MFIQ Score**	**No. of Patients, *n* (%)**			* *p*-Value
		**Trismus** **5 (14.7)**	**No Trismus** **29 (85.3)**	**Total** **34 (100.0)**	
T3	Mean ± Std	10.4 ± 8.2	4.6 ± 8.0	5.4 ± 8.2	39.50 (0.097)
	Median (IQR)	8.0 (14.0)	0 (8.0)	0 (12.3)	
	Min	0	0	0	
	Max	22.0	28.0	28.0	

* Mann–Whitney test.

**Table 6:** The relationship between demographic and clinical variables affecting intervention success.

Demographic/Clinical Information	No. of Patients T1, *n* (%)			*p*-Value	No. of Patients T2, *n* (%)			*p*-Value	No. of Patients T3, *n* (%)			*p*-Value
	Trismus 9 (100.0)	No Trismus 29 (100.0)	Total 38 (100.0)		Trismus 4 (100.0	No Trismus 30 (100.0)	Total 34 (100.0)		Trismus 5 (100.0)	No Trismus 29 (100.0)	Total 34 (100.0)	
Age (years)				0.244 ^a^				0.806 ^a^				0.413 ^a^
Mean ± Std	47.0 ± 16.4	54.2 ± 14.1	52.5 ± 14.8		51.0 ± 18.3	53.4 ± 14.5	53.2 ± 14.7		47.4 ± 17.8	54.1 ± 14.3	53.2 ± 14.7	
Median (IQR)	49.0 (34.0)	55.0 (23.0)	54.0 (25.0)		56.5 (33.0)	55.0 (25.0)	55.0 (24.0)		52.0 (35.0)	55.0 (23.0)	55.0 (24.0)	
Min	25.0	22.0	22.0		25.0	22.0	22.0		25.0	22.0	22.0	
Max	69.0	74.0	74.0		66.0	74.0	74.0		66.0	74.0	74.0	
Gender:				0.128 ^b^				0.274 ^b^				0.348 ^b^
Male	3 (33.3)	19 (65.5)	22 (57.9)		1 (25.0)	20 (66.7)	21 (61.8)		2 (40.0)	19 (65.5)	21 (61.8)	
Female	6 (66.7)	10 (34.5)	16 (42.1)		3 (75.0)	10 (33.3)	13 (38.2)		3 (60.0)	10 (34.5)	13 (38.2)	
Race:				0.761 ^b^				1.000 ^b^				0.175 ^b^
Malay	7 (77.8)	18 (62.1)	25 (65.8)		3 (75.0)	19 (63.3)	22 (64.7)		3 (60.0)	19 (65.5)	22 (64.7)	
Chinese	2 (22.2)	10 (34.5)	12 (31.6)		1 (20.0)	10 (33.3)	11 (32.4)		1 (20.0)	10 (34.5)	11 (32.4)	
Indigenous	0 (0)	1 (3.4)	1 (2.6)		0 (0)	1 (3.3)	1 (2.9)		1 (20.0)	0 (0)	1 (2.9)	
Type of cancer:				0.021 ^b^				0.103 ^b^				0.335 ^b^
Nasopharyngeal carcinoma	1 (11.1)	17 (58.6)	18 (47.4)		0 (0)	17 (56.7)	17 (50.0)		1 (20.0)	16 (55.2)	17 (50.0)	
Non-Nasopharyngeal carcinoma	8 (88.9)	12 (41.4)	20 (52.6)		4 (100.0)	13 (43.3)	17 (50.0)		4 (80.0)	13 (44.8)	17 (50.0)	
Treatment Regimen:				0.260 ^b^				0.039 ^b^				0.164 ^b^
Surgery	6 (66.7)	12 (41.4)	18 (47.4)		4 (100.0)	12 (40.0)	16 (47.1)		4 (80.0)	12 (41.4)	16 (47.1)	
No Surgery	3 (33.3)	17 (58.6)	20 (52.6)		0 (0)	18 (60.0)	18 (52.9)		1 (20.0)	17 (58.6)	18 (52.9)	
TNM Staging:				0.396 ^b^				1.000 ^b^				1.000 ^b^
Stage 1–2	1 (11.1)	9 (31.0)	10 (26.3)		1 (25.0)	9 (30.0)	10 (29.4)		1 (20.0)	9 (31.0)	10 (29.4)	
Stage 3–4	8 (88.9)	20 (69.0)	28 (73.7)		3 (75.0)	21 (70.0)	24 (70.6)		4 (80.0)	20 (69.0)	24 (70.6)	
RT (Gy)				0.304 ^a^								
Mean ± Std	64.0 ± 5.7	64.3 ± 9.5	64.2 ± 8.7		64.5 ± 3.0	63.9 ± 9.5	63.9 ± 9.0	0.420 ^a^	65.6 ± 3.6	63.7 ± 9.7	63.9 ± 9.0	0.772 ^a^
Median (IQR)	66.0 (8.0)	70.0 (10)	66.0 (10.0)		66.0 (5.0)	70.0 (10)	66.0 (10.0)		66.0 (6.0)	70.0 (10.0)	66.0 (10.0)	
Min	52.0	30.0	30.0		60.0	30.0	30.0		60.0	30.0	30.0	
Max	70.0	70.0	70.0		66.0	70.0	70.0		70.0	70.0	70.0	
RT (cycle)								0.338 ^a^				0.682 ^a^
Mean ± Std					32.3 ± 1.5	31.6 ± 6.2	31.7 ± 5.8		32.8 ± 1.8	31.5 ± 6.2	31.7 ± 5.8	
Median (IQR)					33.0 (2.0)	35.0 (5.0)	34.0 (5.0)		33.0 (3.0)	35.0 (5.0)	34.0 (5.0)	
Min					30.0	10.0	10.0		30.0	10.0	10.0	
Max					33.0	35.0	35.0		35.0	35.0	35.0	

^a^ Mann–Whitney test; ^b^ Fisher’s Exact test.

## Data Availability

The data presented in this study are available on request from the corresponding author due to privacy restrictions.

## Supplementary Materials

The following supporting information can be downloaded at: https://www.mdpi.com/article/10.3390/cancers18193215/s1, Table S1: Distribution of non-NPC primary tumour sites by groups; Table S2: Distribution of surgical procedure by groups; Table S3: Surgical procedure of each patient by groups with/without baseline trismus.

## Abbreviations

The following abbreviations are used in this manuscript:

HNC	Head and Neck Cancer
HCTM	Hospital Canselor Tuanku Mukhriz
RT	Radiation Therapy
RIT	Radiation-Induced Trismus
MMO	Maximal Mouth Opening
MID	Maximal Interincisal Distance
Therabite	TheraBite Jaw Motion Rehabilitation System
DTS	Dynasplint Trismus System
MFIQ	Mandibular Function Impairment Questionnaire
SPSS	Statistical Package for the Social Sciences
RCT	Randomised Controlled Trial
RIF	Radiation-Induced Fibrosis
IQR	Interquartile Range
SD	Standard Deviation
IMRT	Intensity-Modulated Radiotherapy
TNM	Tumour–Node–Metastasis
NPC	Nasopharyngeal Carcinoma
3DCRT	Three-Dimensional Conformal Radiotherapy
ARD	Absolute Risk Difference
ISRCTN	International Standard Randomised Controlled Trial Number
