# Supplementary material for: Effect of Jaw Exercise on Mouth Opening and Trismus in Head and Neck Cancer Patients Undergoing Radiotherapy: A Randomised Controlled Trial
Source: Cancers (Basel). 2026 Oct 5;18(19):3215. doi: 10.3390/cancers18193215 (PMC13664613; doi:10.3390/cancers18193215)
Supplement: Supplementary file 1 [file cancers-18-03215-s001.zip › cancers-4546540-supplementary.pdf]

Table S1: Distribution of non NPC primary tumour sites by groups

Tumour Site	No of patients *n* (%) Intervention 10 (100.0)	Control 10 (100.0)	Total 20 (100.0)
Oral and oropharynx carcinoma/angiosarcoma	4 (40.0)	6 (60.0)	10 (50.0)
Salivary gland carcinoma	4 (40.0)	3 (30.0)	7 (35.0)
Paranasal carcinoma	1 (10.0)	0 (0)	1 (5.0)
Temporal bone carcinoma	1 (10.0)	1 (10.0)	2 (10.0)

Table S2: Distribution of surgical procedure by groups

Surgical Procedure	Type	No of patients *n* (%)		Total 18 (100.0)
		Intervention 8 (100.0)	Control 10 (100.0)	
Mandible/Maxilla	Mandibulotomy/Mandibulectomy/ Maxillectomy	3 (37.5)	3 (30.0)	6 (33.3)
	No Mandibulotomy/Mandibulectomy/ Maxillectomy	5 (62.5)	7 (70.0)	12 (66.7)
Reconstructive	Primary closure	6 (75.0)	6 (60.0)	12 (66.7)
	Locoregional flap	1 (12.5)	2 (20.0)	3 (16.7)
	Free Flap	1 (12.5)	2 (20.0)	3 (16.7)
Neck Dissection	Neck Dissection	5 (62.5)	8 (80.0)	13 (72.2)
	No Neck Dissection	3 (37.5)	2 (20.0)	5 (27.8)

Table S3: Surgical procedure of each patient by groups with/without baseline trismus

No	Surgical Procedure	Group	Baseline Trismus ≤35mm
1	• Tracheostomy<br>• Wide local excision<br>• Partial resection of left parotid and lower 2 branches of facial nerve<br>• Left modified radical neck dissection<br>• Left hemi- mandibulectomy<br>• Reconstruction with osteocutanous fibular and anterolateral thigh free flap	Intervention	Yes
2	• Left subtotal maxillectomy with obturator	Intervention	Yes
3	• Tracheostomy<br>• Subtotal glossectomy via lip split mandibulotomy<br>• Left modified radical neck dissection & right selective neck dissection<br>• Reconstruction with pectoralis major myocutaneous flap	Intervention	Yes
4	• Wide local excision + primary closure<br>• Right selective neck dissection	Intervention	No
5	• Left superficial parotidectomy<br>• Excision biopsy of left level II lymph node	Intervention	No
6	• Right completion parotidectomy<br>• Selective right neck dissection	Intervention	No
7	• Left partial glossectomy + primary closure<br>• Left selective neck dissection	Intervention	No
8	• Left superficial parotidectomy	Intervention	No
**No**	**Surgical Procedure**	**Group**	**Baseline Trismus ≤35mm**
**1**	• Tracheostomy<br>• Wide local excision<br>• Right marginal mandibulectomy<br>• Right modified radical neck dissection<br>• Reconstruction with right masseteric myofascial pedicled flap, buccal fat pad and free transfer skin graft	Control	Yes
**2**	• Left total parotidectomy with facial nerve preservation<br>• Selective left neck dissection	Control	Yes
**3**	• Left submandibulectomy<br>• Selective left neck dissection	Control	Yes
4	• Right partial parotidectomy<br>• Right radical neck dissection<br>• Reconstruction with pectoral major myofascial flap<br>• Bilateral Tonsillectomy	Control	No
5	• Wide local excision + primary closure	Control	No

	• Right selective neck dissection		
6	• Tracheostomy<br>• Subtotal glossectomy via lip split mandibulotomy<br>• Bilateral Selective Neck Dissection<br>• Reconstruction with left radial forearm free flap + full thickness split skin graft from left groin	Control	No
7	• Posterior left cortical mastoidectomy	Control	No
8	• Left middle meatal antrostomy + posterior septectomy + nasopharyngectomy with tumour debulking	Control	No
9	• Tracheostomy,<br>• Bilateral selective neck dissection<br>• Subtotal glossectomy via lip split mandibulotomy<br>• Reconstruction with left anterolateral thigh flap	Control	No
10	• Right submandibulectomy<br>• Right radical neck dissection	Control	No
